# Clustering Analysis Algorithm of Volleyball Simulation Based on Radial Fuzzy Neural Network

**DOI:** 10.1155/2022/8365024

**Published:** 2022-05-17

**Authors:** Xiaoshen Yan, Yu Wang

**Affiliations:** ^1^Department of Sports, Zhongnan University of Economics and Law, Wuhan, Hubei 430073, China; ^2^Office of Academic Research, Changjiang Polytechnic, Wuhan, Hubei 430074, China

## Abstract

Aiming at a series of problems existing in volleyball, based on radial fuzzy neural network theory, the optimized simulation clustering analysis algorithm is used to monitor and analyze volleyball. By analyzing the feature weights of nodes at different stages, the optimal radial fuzzy neural network was constructed, which was combined with the simulation clustering algorithm to obtain the relevant optimization model describing volleyball. The accuracy of the optimization model is verified by comparing with the original model. The results show that with the increase of response, the response curves of different algorithms show fluctuation. Among them, the fluctuation range of MPDR (minimum power distortionless response) algorithm is larger, and the value of the curve obtained by MVDR (minimum variance distortionless response) algorithm differs greatly from that of the optimization algorithm at some key nodes, while the beam changing chart obtained by the optimization algorithm can better reflect the changing trend of the beam. Model indexes under different algorithms are different. When the number of iterative steps is less than 30, indexes under different algorithms are all greater than the standard value. When the number of iterations is more than 30, the indexes under different algorithms are all less than the standard value. Through verification, it can be seen that the original model can only describe the first stage of volleyball, while the optimization model can describe the whole process of volleyball. It shows that the optimization model can be used to describe and analyze volleyball-related data. And the algorithm can be used to better predict and analyze volleyball, and the analysis results can provide relevant guidance for volleyball. The optimization model provides basis and theoretical support for the application of volleyball simulation clustering algorithm, so as to better promote volleyball and better guide the movement.

## 1. Introduction

Volleyball is an important part of sports. The existing volleyball has some typical problems including insufficient venue facilities and the lack of professionals. The most important factor is the lack of the analysis and guidance of the corresponding algorithm in the development process of the existing volleyball so that the development of volleyball is limited, but at present, fuzzy neural network and simulation clustering algorithms are commonly used to solve the practical problems [1–3].

Radial fuzzy neural network has been widely used in the fields of pattern recognition, simulation analysis, and image processing. In order to improve the performance of shunt filter, an adaptive fuzzy neural network control scheme based on radial basis function neural network was proposed [4]. Compared with traditional passive filter, this optimization scheme can eliminate harmonic pollution better and further improve mechanical power. Aiming at a series of problems existing in the application of the original fuzzy neural network, an improved version based on radial basis function neural network was proposed [5]. This model can effectively avoid the influence on the output layer in the calculation process, so as to improve the accuracy of data use. Moreover, the optimized clustering algorithm does not depend on parameter adjustment, and the accuracy of the model is verified by using relevant data and cases. The adaptive fuzzy radial basis function neural network is adopted to optimize the traditional model, and a new model is obtained [6]. By comparing the simulation results of the optimized model with the traditional control method, it can be seen that the optimized fuzzy neural network control method can further reduce the maximum error of the model, thus providing theoretical knowledge and analysis for the application of intelligent algorithm in power system. In order to effectively combine useful information with image details, a new image fusion method is proposed [7]. By using the method of fuzzy neural network, the details or feature information of different images is fully analyzed based on the simulation clustering analysis algorithm. Finally, the fused images are obtained by using the inverse wavelet transform technology. The optimized simulation method is verified by laboratory test, and the superiority of the model is demonstrated. The method can provide theoretical basis for the application of fuzzy neural network.

Fuzzy neural network (FNN) is a reliable and efficient computing model, which can realize corresponding functions on the microprocessor. An adaptive neural network based on wavelet pattern is compared with clustering methods such as radial basis function network [8]. By optimizing the structural parameters of all the prediction models to produce the most efficient structure, then, by comparing the mean square error, calculation time, and prediction process of the optimized model, the corresponding guidance can be provided for the improvement of the adaptive model through comparative analysis. An enhanced fuzzy radial basis function neural network model was proposed [9], which includes input layer, hidden-layer neuron, output layer, and other components. Based on fuzzy neural network theory, the input variables of neural network are selected and analyzed by using correlation matching method. Relevant laboratory tests were carried out to verify the accuracy of the optimization model, and the results show that the data and images obtained by the model are better than other methods, thus obtaining the highest image similarity.

Correlation algorithms of simulation cluster analysis have been widely applied in different fields: a flexible machine with high shear and low pressure was developed [10]. Euler–Lagrange coupling method was adopted to simulate the new pressure-shear process. The results show that the simulation algorithm based on radial fuzzy neural network theory has a good application in fluid, and the ratio of axial tangential force to radial normal force is improved compared with traditional pressure shear. Thus, the effectiveness of high-shear low-pressure grinding is further verified. Based on the principle of fuzzy neural network, an optimized clustering method based on the spatial distribution of ground motion is applied to simulate the propagation of seismic waves [11], so as to further obtain relevant ground motion data. The seismic source model and 3D velocity structure model are constructed by theoretical analysis and numerical calculation. In order to study the variation of battery parameters in transient one-dimensional simulation, three reduced order models [12] were proposed according to the relevant characteristics of battery parameters: appropriate orthogonal decomposition model, simulation cluster analysis model, and orthogonal cluster analysis model.

Simulation clustering analysis not only has a good application in industry but also can be applied to students' classroom: for the current students' classroom behavior recognition, algorithm has a series of problems such as insufficient accuracy. The traditional clustering analysis algorithm and fuzzy neural network algorithm are combined to improve the traditional algorithm [13], and the real-time identification and monitoring of students' activities is carried out by combining the relevant optimization model. In order to verify the accuracy of the model, relevant experiments were carried out by extracting the relevant parameters of students' classroom spatial angle features. Relevant studies show that the fuzzy neural network structure is superior to the network structure with single feature. A simplified clustering analysis ionization method based on the simulation clustering analysis method under radial fuzzy neural network was used to estimate the electron and related gamma radiation of complex DNA damage [14]. The accuracy of the optimized model was verified by laboratory experiments, and the study showed that the estimation model was helpful to study the complexity of DNA damage after radiation and electron irradiation.

Many experts and scholars have carried out relevant algorithm research on volleyball: simulated volleyball match is a phenomenon that has emerged in recent years, and match between teams can be simulated by using algorithms. An improved sine and cosine algorithm was proposed [15]. Using this algorithm, a more accurate solution can be obtained. By using SCA operator, a more efficient method to find the optimal solution of the optimization problem can be obtained. The results show that the proposed algorithm has better performance than other algorithms. As a new content in the field of computer vision, object tracking can be used to study volleyball players' motion state in the testing stage [16]. Volleyball is normalized and three different metaheuristic algorithms are used to track volleyball, so as to get the relevant laws of volleyball. Due to the small size of volleyball, the speed of volleyball movement will be blurred, resulting in the old system difficult to analyze the volleyball level. Volleyball processing speed is improved by using machine vision and wearable devices to track volleyball movement [17]. Relevant studies show that the volleyball motion estimation algorithm is correct and its simulated trajectory is almost consistent with that of volleyball. The above research failed to carry out corresponding research on volleyball. In view of the shortcomings of the above research, this paper, based on the relevant theory of radial fuzzy neural network, adopts the simulation clustering analysis algorithm to carry out quantitative research on the relevant indicators of volleyball. On the basis of the study of relevant indicators, the corresponding optimization model was obtained by modifying the relevant parameters of the original model, and the superiority of the model was verified by relevant calculation. The research can provide theoretical basis for the application of radial fuzzy neural network and simulation clustering analysis algorithm. Therefore, it can provide corresponding guidance for the development of volleyball. The research shows that the model can not only be used for volleyball but also has a good description for other kinds of sports. Therefore, we can further promote the optimization model on the basis of the research in this paper.

## 2. Radial Basis Function (RBF) Neural Network

### 2.1. Radial Neural Network

**Figure 1:**
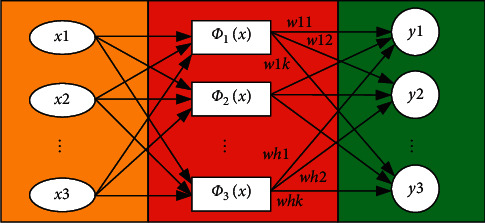
Structure of radial function neural network.

Radial basis function (RBF) neural network is a kind of forward network. It has the advantages of simple training, fast learning convergence, and overcoming local minimum problem [18]. It has been proved that RBF networks can approximate any continuous function with any accuracy. Therefore, it has been widely used in pattern recognition, nonlinear control, and image processing. The structural of radial basis function neural network is shown in Figure 1. Input value xi is imported into the corresponding function *φ*i(x), respectively, and the corresponding function yi value is output [19].

In RBFNN (radial basis function neural network), the RBF is usually taken as the transfer function, the parameters of vector control are taken as parameters of RBF, and the distance between the input sample and the hidden center vector is taken as the mapping of variables. The corresponding formula is shown as follows:

(1) $$\begin{array}{c} \phi_{j}\left(x\right)=K\left(\left\|x-c_{j}\right\|,\sigma_{j}\right)\;j=1,2,\ldots ,m, \end{array}$$

where the *c*_*j*_ is the hidden center vector; *σ*_*j*_ is the kernel function control parameter; ||*x*−*c*_*j*_|| is the distance between *x* and *c*_*j*_; and *m* represents the number of nodes in the hidden layer.

(2) $$\begin{array}{c} R_{j}\left(x\right)=\exp\left[-\frac{{\left\|x-c_{j}\right\|}^{2}}{2\sigma_{j}^{2}}\right]\;j=1,2,\ldots ,m. \end{array}$$

The linear combination of the RBF function *φ*_*j*_(*x*) in the hidden layer node forms the output *y*_*k*_ of RBFNN

(3) $$\begin{array}{c} y_{k}=\sum_{j=1}^{m}w_{jk}\phi_{j}\left(x\right)\;k=1,2,\ldots ,p, \end{array}$$

where the *w*_*jk*_ represents the connection weight of the *k*-th node in the output layer and the *j*-th node in the hidden layer; *y*_*k*_ represents the output of the *k*-th node in the output layer; and *p* is the number of output nodes.

The minimum variance distortionless response (MVDR) algorithm and the minimum power distortionless response (MPDR) algorithm modify the covariance matrix by using the convex linear combination of the unit matrix and the sampling covariance matrix. On this basis, fuzzy radial basis (RBF) neural network approximation algorithm is introduced to obtain the optimization model, and the nonlinear mapping from array covariance matrix to optimal weight vector is realized through the optimization model.

**Figure 2:**
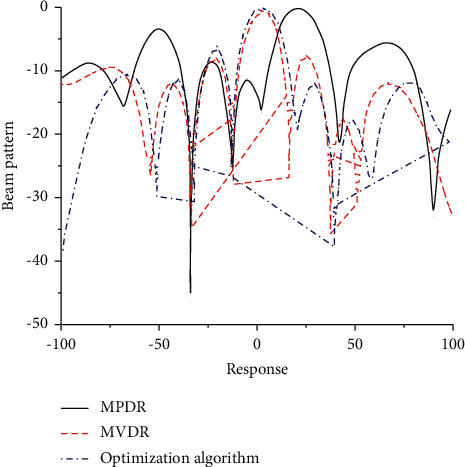
Response diagram of different algorithms.

Through the analysis of different algorithms in the fuzzy neural network, response diagrams of corresponding algorithms are obtained, as shown in Figure 2. As can be seen from Figure 2, the change curves of different algorithms as a whole show the change characteristics of multiple fluctuations. The fluctuation amplitude of MPDR algorithm is relatively obvious. With the corresponding gradual increase, the correlation between the corresponding beam change and the optimization algorithm is poor, which cannot reflect the change characteristics of the beam better. The MVDR algorithm is close to the wave characteristics of the optimization algorithm on the whole, but the value of the optimization algorithm differs greatly from that of the optimization algorithm at some key nodes. Therefore, neither MPDR nor MVDR algorithm can better describe the corresponding beam change trend, while the corresponding optimization algorithm can better reflect the beam change trend. The main reasons for MVDR algorithm not being able to describe and represent the beam graph well in the calculation process include the following: (1) the number of iterations of this algorithm is relatively small in the calculation process, and the smaller the number of iterations is, the larger the deviation of the calculation results will be; (2) at the same time, the algorithm is subjected to a large amount of drying in the operation process, which makes the MVDR algorithm that has deeper defects in the interference direction, leading to the deviation of results; (3) and no corresponding inverse operation is also the main reason for the deviation of MVDR algorithm.

### 2.2. Probabilistic Neural Network

**Figure 3:**
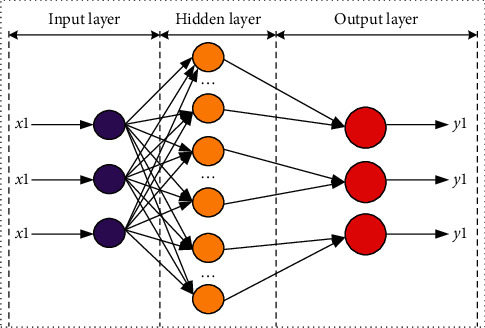
Structure of probabilistic neural network.

Probabilistic neural network is usually composed of three layers, namely, input layer, hidden layer, and output layer [20]. Its structural model is shown in Figure 3.

Assuming *x* is the *n*-dimensional input sample and the transfer function of the hidden layer node is K, then the output *y*_*k*_ of the k-th node of PNN can be expressed as follows:

(4) $$\begin{array}{c} y_{k}=\frac{1}{H}\sum_{j=1}^{H_{k}}K\left(\left\|x-c_{j}^{k}\right\|,\sigma \right), \end{array}$$

where the *c*_*kj*_ represents the *j*-th hidden node corresponding to the *k*-th category; *σ* is the function control parameter; *H*_*k*_ represents the number of hidden nodes corresponding to the *k*-th category in the probabilistic neural network; and *K* is the basis function. The output value of the *k*-th node is as follows:

(5) $$\begin{array}{c} y_{k}=\frac{1}{{\left(2\pi \right)}^{m/2}\sigma^{m}H_{k}}\sum_{j=1}^{H_{k}}\exp\left(-\frac{{\left\|x-c_{j}^{k}\right\|}^{2}}{\sigma }\right)=\frac{1}{{\left(2\pi \right)}^{m/2}\sigma^{m}H_{k}}\sum_{j=1}^{H_{k}}\exp\left(\frac{x^{T}c_{j}^{k}-1}{\sigma^{2}}\right), \end{array}$$

where the *m* is the spatial dimension of input sample *x*.

**Figure 4:**
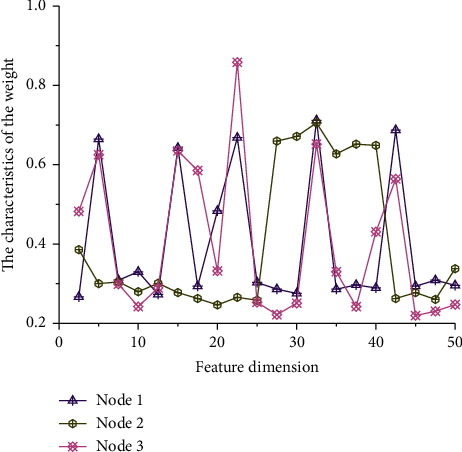
Distribution of characteristic weights of simulated data at different nodes.

Through the above analysis, the distribution of characteristic weights of simulated data at different nodes is drawn, as shown in Figure 4. As can be seen from Figure 4, with the increase of feature dimension, the curve of node 1 shows a trend of fluctuation on the whole, the lowest feature weight is 0.267, the highest feature weight is 0.712, and the highest value is 2.67 times of the lowest value. It shows that the feature dimension has a great influence on the feature weight of node 1, and the curve fluctuates, which is detrimental to the overall stability of the model. With the increase of feature dimension, the overall change curve of node 2 can be divided into three stages: (1) the stable stage with feature dimension between 0 and 25 in which the feature weight is generally low; (2) the fluctuation stage of feature dimension between 25 and 42 and the corresponding feature weight of this stage are generally higher; and (3) the steady increase stage of feature dimension between 42 and 50, and the range of feature weights in this stage are small. The variation rule of feature weights corresponding to node 3 is basically the same as that of node 1, except that there are maximum and minimum values of feature weights corresponding to node 3. The main reasons for the deviation of feature weights of different nodes are as follows: (1) the input values at different nodes are different, which leads to different dimensions of input samples. The different dimensions change the transfer function and finally lead to different output results. (2) The sample space corresponding to different nodes is different, which changes the hidden layer and output layer of the corresponding model, and ultimately affects the difference of feature weights, leading to the deviation of feature weights of different nodes.

### 2.3. Radial Basis Probabilistic Neural Networks

Radial basis probabilistic neural network absorbs the advantages of radial basis function neural network and probabilistic neural network; that is, it takes full account of the staggered influence between multiple types of patterns in the application of pattern recognition, thus forming an effective interface. In particular, this new model has the advantages of low computational complexity and fast convergence. At the same time, it is also a new neural network model, which can be widely used in pattern recognition and nonlinear function approximation. The training specimens of radial basis probabilistic neural network (RBPNN) can fully consider the influence of different modes [21]. The model structure can be divided into input layer, first hidden layer, second hidden layer and output layer, respectively [22]. *x* is an *n*-dimensional input sample, and the transmission function of the first hidden node is *K*. The specific calculation formula of output *z*_*i*_ corresponding to the *i*-th node is as follows:

(6) $$\begin{array}{c} z_{i}=K\left(\left\|x-c_{j}\right\|,\sigma \right)\;i=1,2,\ldots ,n_{1}, \end{array}$$

where *n*_1_ represents the number of nodes of the first hidden layer, and the specific formula is as follows:

(7) $$\begin{array}{c} r_{i}=\exp\left[-\frac{\left\|x-c_{j}\right\|}{2\sigma^{2}}\right]\;j=1,2,\ldots ,n_{1}. \end{array}$$

The connection weight *w*_*i*_ (2) of the second hidden layer is selected as 1, and then, the output *u*_*j*_ of the *j*-th node of the second hidden layer is calculated by the following formula:

(8) $$\begin{array}{c} u_{j}=\sum_{i=1}^{H_{j}}K\left(\left\|x-c_{j}\right\|,\sigma \right)\;k=1,2,\ldots ,n_{2}, \end{array}$$

where *n*_2_ represents the number of nodes of the second hidden layer, and *H*_*j*_ represents the number of *j*-th nodes in the second hidden layer.

The fourth layer of RBPNN model is the output layer, which is a linear combination of the outputs of nodes of the second hidden layer. The specific calculation formula of the output *y*_*k*_ of the *k*-th node is as follows:

(9) $$\begin{array}{c} y_{k}=\sum_{j=1}^{n_{2}}w_{j}^{\left(3\right)}u_{j}\;k=1,2,\ldots ,p, \end{array}$$

where the *p* is the number of nodes in the output layer. *w*_*j*_ (3) is the connection weight between the output layer and the second hidden layer.

(10) $$\begin{array}{c} d_{i}\left(t\right)=\left\|x\left(t\right)-c_{i}\left(t-1\right)\right\|,1\leq i\leq M, \end{array}$$

where *x*(*t*) is the input vector, and *c*_*i*_(0) is the cluster center.

(11) $$\begin{array}{c} \sigma_{i}=\frac{d_{m}}{\sqrt{2M}}, \end{array}$$

where *d*_*m*_ = max(*d*_*i*_(*t*)) and *M* represents the number of nodes of the first hidden layer.

(12) $$\begin{array}{c} z_{i}\left(x_{t}\right)=\sum_{l=1}^{n_{t}}\phi \left(x_{t},c_{l}^{i},\sigma \right)=\sum_{l=1}^{n_{t}}\phi \left(\left\|x_{t}-c_{l}^{i}\right\|,\sigma \right),\;i=1,2,3,\ldots ,p. \end{array}$$

**Figure 5:**
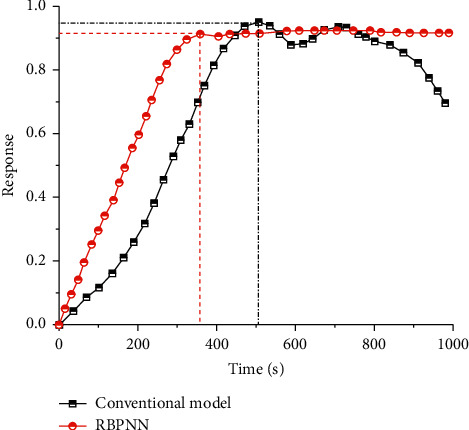
Signal output response of different models.

Through the above analysis, the time-varying curves of signal output response in the conventional model [8] and RBPNN [23] model were drawn, as shown in Figure 5. The output influence of the conventional model can be divided into two stages [24]. The first stage is the stable growth stage, with the increase of time, and the corresponding signal output influence increases with the change trend of approximately linear increase. The second stage is the fluctuation, with the further improvement of time, and the corresponding change trend is shown as fluctuation, indicating that the output signal of the model is unstable. It is worth noting that the critical value of the two stages is the maximum signal output response of the model. The response change rule of RBPNN model can also be divided into two stages. The first stage shows an approximate linear change trend. Compared with the conventional model, the slope of the response curve of RBPNN model is higher, indicating that the response changes with time are more relevant. In the second stage, the overall change trend is similar to the level. Compared with the fluctuation change of the conventional model, the model has better stability. In general, RBPNN model has better stability and superiority than conventional model. The main reasons for the deviation of the conventional model are as follows: (1) the model does not take into account the interlacing effect among multiple types of patterns in the application of pattern recognition, and the recognition of multiple patterns is insufficient; (2) at the same time, the conventional model lacks effective interface and analysis of corresponding signal classification; and (3) finally, compared with the optimization model, the calculation of the conventional model is more complex, which leads to a slower convergence rate.

**Figure 6:**
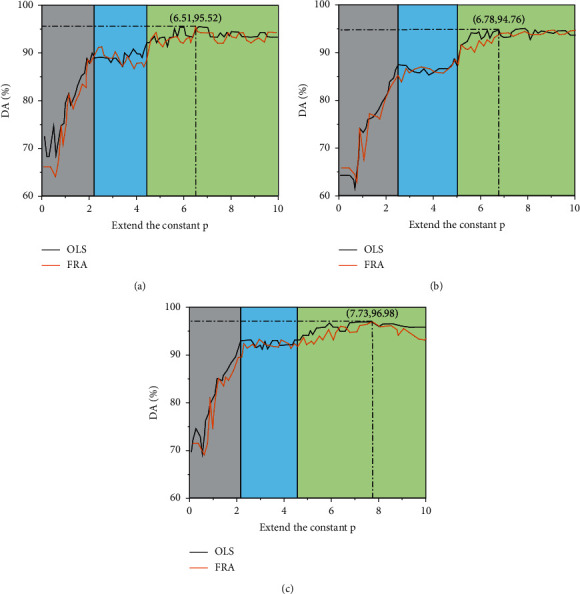
Extended constant optimization diagram under different neural networks. (a) Neural network NN1. (b) Neural network NN2. (c) Neural network NN3.

In order to explore the computing accuracy under different neural networks, the variation relationship between the expansion constant P and the diagnostic accuracy (DA) of different neural networks was drawn, as shown in Figure 6, and the pairs of different neural networks are shown in Table 1. It can be seen from Figure 6 that the change curves between the expansion constant and accuracy rate under different neural networks can be divided into three stages. The first stage is the rapid increase stage in which the change curves of orthogonal least squares (OLS) and fast recursive algorithm (FRA) [25] both show rapid increase with the increase of the expansion constant. The corresponding second stage is mainly represented by fluctuations within a certain range, indicating that the accuracy rate has poor stability within the range of this extended constant. In the third stage, the overall variation range of accuracy is small, indicating that the accuracy has a high stability under the extended constant, and the variation trend of the two algorithms is basically the same.

It can be seen from Table 1 that the accuracy of NN3 is the highest, that of NN1 is in the middle, and that of NN2 is the lowest, indicating that the accuracy of corresponding models varies under different neural networks. And the optimal expansion constant is also different with the change of neural network. When the neural network is NN3, the extension constant obtained by OLS algorithm is the largest, while when the neural network is NN1, the extension constant obtained by FRA algorithm is the smallest, and the maximum value is about 1.21 times of the minimum value.

## 3. Simulation Cluster Analysis

### 3.1. Clustering Analysis

**Figure 7:**
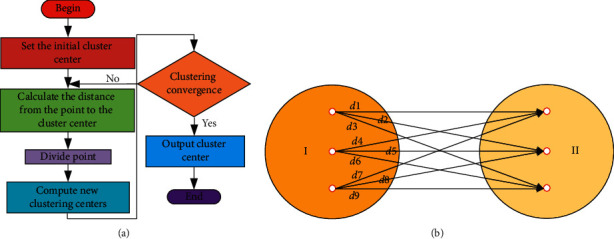
Cluster analysis. (a) Flow chart and (b) schematic diagram.

Cluster analysis refers to the process of grouping data by analyzing the characteristics of each data for a batch of unknown data sets and aggregating data with similar or identical characteristics. The main process of cluster analysis can be divided into the following four steps [26, 27]:

(1) Data standardization processing:
The purpose of data standardization is to prevent the existence of large data or data with different units in data:
  (13) $$\begin{array}{c} x_{{}_{ij}}^{*}=\left\{\begin{array}{c} \frac{x_{ij}-{\bar{x}}_{j}}{S_{j}},S_{j}\neq 0,\;i=1,2,3,\ldots ,n,\\ 0,S_{j}=0,\;j=1,2,3,\ldots ,m, \end{array}\right. \end{array}$$
where the S is the standard deviation and ${\bar{x}}_{j}$ is the mean value.
(2) Construct the relation matrix:
Using the index of squared Euclidean distance to construct the relation matrix of standardized data:
  (14) $$\begin{array}{c} d\left(x,y\right)=\sum_{i}{\left(x_{i}-y_{i}\right)}^{2},\\ J\left(c,\mu \right)={\sum_{i=1}^{k}\left\|x^{\left(i\right)}-\mu_{c\left(i\right)}\right\|}^{2}. \end{array}$$
In the formula, (*i*) represents the mean value of the *i*-th cluster.
(3) Cluster analysis based on different methods:
Cluster analysis methods can be divided into systematic clustering method and K-means clustering method. The k-means algorithm first needs to determine the number of clusters and the center point of the initial cluster, and then completes the division after several iterations by calculating the distance from the center point:
  (15) $$\begin{array}{c} S=\sum_{i=1}^{n}\underset{j\in \left\{1,\ldots ,k\right\}}{\min}{\left|x_{i}-a_{j}\right|}^{2}. \end{array}$$
Figure 7 is the flow chart and schematic diagram of cluster analysis method.  Figure 7(a) is the data processing flow chart of cluster analysis. Firstly, the corresponding initial cluster center shall be set for the model. Secondly, the distance between the correlation points and the cluster center shall be calculated, and the correlation points shall be divided according to the calculated distance. According to the partition results, a new cluster center is set up to verify whether the cluster center converges, and finally, the relevant data are output.  Figure 7(b) shows the schematic diagram of cluster analysis, where 3 elements in category 1 and 3 elements in category 2 coexist *d*_1_, *d*_2_,…, *d*_9_. The distance between class 1 and class 2 can be calculated as follows:
  (16) $$\begin{array}{c} \bar{d}=\frac{d_{1}+d_{2}+\cdots +d_{9}}{9}, \end{array}$$
(4) Determine the best classification:

After the systematic clustering method, the clustering tree graph can be obtained, and the clustering results can be analyzed and processed according to the relevant criteria.

### 3.2. Fuzzy Clustering Analysis

The basic principle of fuzzy mean clustering analysis is an algorithm that quantitatively describes the membership function of each sample belonging to a certain category in the absence of relevant data. The fuzzy partition space of *X* is shown as follows:

(17) $$\begin{array}{c} M_{fc}=\left\{U\in R^{cn}|\mu_{ik}\in \left[0,1\right],\forall i,k;\sum_{i=1}^{c}\mu_{ik}=1,\forall k\right\}, \end{array}$$

where the *U* = [*μ*_*ik*_]*c* × *n* is the fuzzy partition matrix, *μ*_*ik*_ represents the fuzzy membership degree of sample *x*_*k*_ belonging to class *i*, *i* is 1 as fault class, and *i* is 2 as nonfault class.

The value function based on dissimilarity index is shown as follows:

(18) $$\begin{array}{c} \left\{\begin{array}{c} J_{m}=\left(U,P\right)=\sum_{k=1}^{n}\sum_{i=1}^{c}{\left(\mu_{ik}\right)}^{m}{\left\|x_{k}-p_{i}\right\|}^{2},m\in \left[1,\infty \right),\\ s.t.U\in M_{fc}, \end{array}\right. \end{array}$$

where *P* is *c* cluster center vectors, and *m* is weighted index.

The minimum value of value function of dissimilarity index is shown as follows:

(19) $$\begin{array}{c} F=\sum_{i=1}^{c}{\left(\mu_{ik}\right)}^{m}{\left\|x_{k}-p_{i}\right\|}^{2}+\lambda \left(\sum_{i=1}^{c}\mu_{ik}-1\right). \end{array}$$

The iterative formula of fuzzy membership degree and clustering center is shown as follows:

(20) $$\begin{array}{c} \mu_{ik}=\frac{{\left(1/{\left\|x_{k}-p_{i}\right\|}^{2}\right)}^{1/\left(m-1\right)}}{\sum_{i=1}^{c}{\left(1/{\left\|x_{k}-p_{i}\right\|}^{2}\right)}^{1/\left(m-1\right)}},\;1\leq i\leq c,\;1\leq k\leq n, \end{array}$$

**Figure 8:**
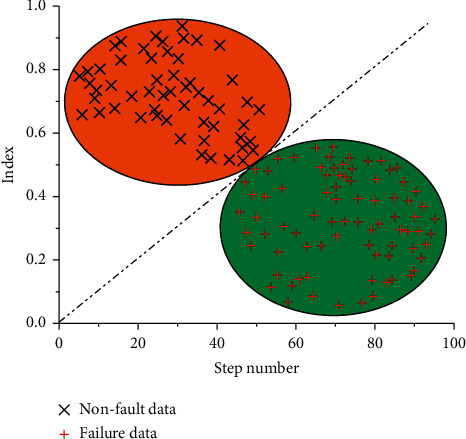
Schematic diagram of principle.

Figure 8 is a schematic diagram of fuzzy clustering analysis. It can be seen from Figure 8 that fuzzy clustering analysis method can make statistics and analysis of fault data and nonfault data, so as to divide nonfault region and fault region. Calibrate the set of fault data and nonfault data, import the calibrated data into the fuzzy neural network, obtain the output model value through the relevant calculation of the fuzzy neural network model, and then compare the output data with the evaluation index, so as to divide the fault data and nonfault data.

**Figure 9:**
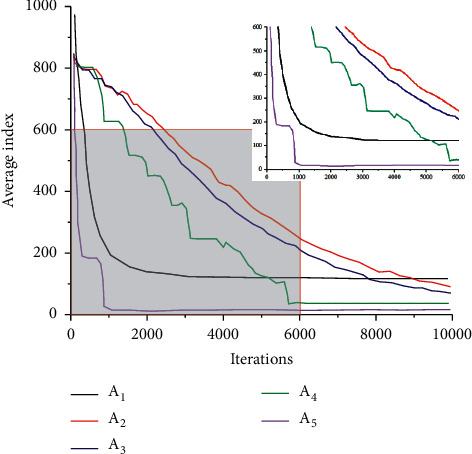
Clustering performance analysis of data.

A1-A5 are all different parameters of fuzzy clustering analysis, to explore the influence of different parameters on fuzzy clustering analysis. The clustering performance analysis graph of data with different parameters was drawn, as shown in Figure 9.

As the number of iterations increased, the overall curve showed a trend of gradual decline. The variation patterns of A2 and A3 curves are basically the same, showing a slow decline. A4 shows a step-down trend, indicating that the number of iterations shows typical nonlinear characteristics to the average index under this parameter. The downward trend of A1 is similar to the change trend of logarithmic function, indicating that there is a high logarithmic function relationship between the number of iterations and the corresponding average index. The curve corresponding to A5 experienced a drop in a short period of time, and then, with the increase of times, the curve was approximately flat, with a small variation range.

## 4. Simulation Analysis of Volleyball

### 4.1. Introduction to Volleyball

Volleyball, as an important part of sports, is an indispensable part of the development of sports, but the existing volleyball has some typical problems:

#### 4.1.1. Lack of Venues and Facilities

With people's love for sports, more and more people are involved in sports. But with the gradual increase in the number of the original volleyball venues and equipment cannot meet the corresponding needs, resulting in people's enthusiasm for physical exercise has been weakened, and the lack of volleyball venues facilities is the main reason for restricting volleyball.

#### 4.1.2. Lack of Professionals

Volleyball is a sport with high risk and may be injured to varying degrees in the process of sports. But at present, the lack of professional volleyball personnel limited the further popularization of volleyball.

#### 4.1.3. Lack of Corresponding Algorithm Analysis

At present, a series of problems in volleyball are mainly studied by qualitative analysis. For the problems existing in volleyball, only from the perspective of qualitative analysis cannot solve the problem.

### 4.2. Clustering Analysis of Volleyball Simulation

**Figure 10:**
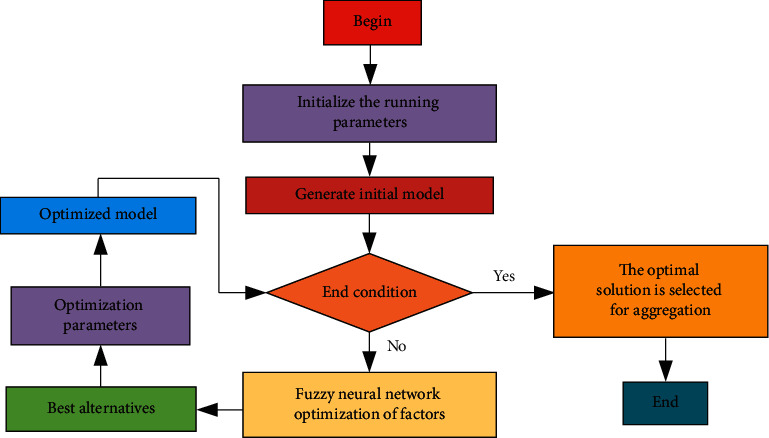
Flow chart of volleyball simulation analysis based on fuzzy neural network.

In order to better study volleyball-related problems, based on the radial fuzzy neural network theory, the simulation clustering analysis algorithm is used to study volleyball. The initial analysis of the relevant parameters of the model is still carried out first, and then, the relevant theoretical model is obtained. If the model does not meet the relevant requirements, it is necessary to optimize the relevant factors in the model, so as to select the optimal scheme and parameters, and finally get the relevant optimization model, and analyze the optimal solution of the model, as shown in Figure 10. The main monitoring indicators include speed, strength, endurance, and flexibility.

Through the correlation analysis of volleyball sports, the distribution table of related attributes of volleyball sports system is obtained, as shown in Table 2.

### 4.3. Application of Cluster Analysis in Volleyball

**Figure 11:**
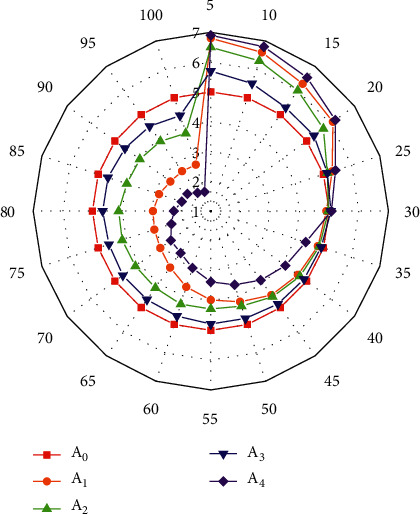
Radar diagram of different algorithms.

In order to better explore the application of different algorithms in the optimization model in volleyball, radar charts of model indicators under different algorithms are drawn, as shown in Figure 11, where A0 is the calculated value of the model and A1-A4 are the model parameters in Figure 9. It can be seen from Figure 11 that model indexes under different algorithms are different. When the number of iterative steps is less than 30, indexes under different algorithms are all greater than the standard value. A4 is the largest, indicating that A4 deviates most from the standard value, followed by A1, A2 is closer to the standard value, and A3 is the algorithm closest to the standard value. When the number of iterations exceeds 30, the indexes under different algorithms are all less than the standard value. A4 is the smallest, indicating that A4 has the largest error, followed by A1. A2 is also close to the standard value, while A3 is closest to the standard value. And A4's algorithm has the lowest accuracy.

**Figure 12:**
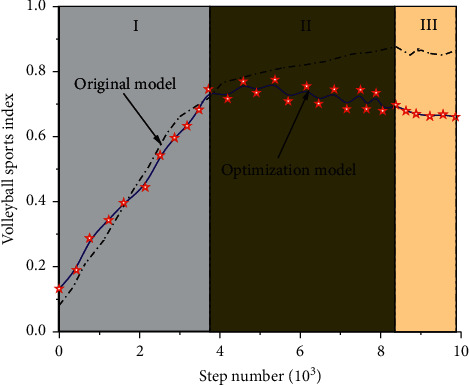
Comparison of different models.

By adopting the above analysis method and based on the simulation clustering analysis algorithm under the radial fuzzy neural network, volleyball movement was analyzed by using the optimized calculation method, so as to obtain the comparison diagram of the theoretical optimization model and the original model under different calculation time, as shown in Figure 12.

As can be seen from Figure 12, as the number of iterative steps increases, the description of data in the original model can be divided into three stages. The first stage is a linear increase stage, and the fitting coefficient of this stage is 0.96, indicating that the original model can be used in the first stage to better describe and analyze volleyball. In the second stage, the original model still showed a trend of linear increase. Compared with the first stage, the increase rate in the second stage decreased. However, the actual data showed a gradual decline and fluctuated to a certain extent. At this stage, it was obvious that the original model could not describe the data, and the correlation between the two was poor. From the third stage, the original model and volleyball-related data show stable changes, but due to the deviation of the original model in the second stage, the deviation between the model and the actual data in the third stage is still large; therefore, the model cannot be used to analyze volleyball-related data. From the perspective of the optimization model, the fitting coefficient of the optimization model to the test data in the first stage is 0.985, indicating that the model can describe the first stage of the curve well. And the optimization model for the second stage of the fluctuation data description result is still very good, and the third stage of the optimization model curve also showed stable changes, so the optimization model can achieve the description and analysis of volleyball-related data.

**Figure 13:**
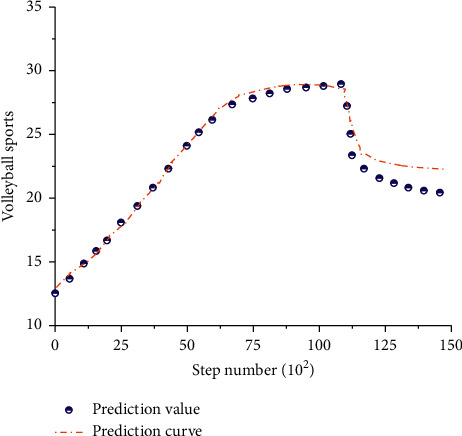
Volleyball prediction chart.

It can be seen from the above analysis that the model obtained by the simulation clustering analysis algorithm optimized based on radial fuzzy neural network can well represent the change process and corresponding change rules of volleyball. At the same time, in order to better predict the relevant indicators of volleyball, volleyball variation curves under different iterations were drawn, as shown in Figure 13.

It can be seen from Figure 13 that the optimization model can better reflect the relevant indicators in volleyball. It can be seen from the curve that volleyball has a linear increase to some extent in a period of time, but when the number of iterations exceeds 7500, the increase of volleyball indicators will significantly decrease. With the further increase of the number of iterations, the corresponding index will drop suddenly and then slowly, indicating that volleyball will decline after a certain period of time. And we should make a detailed analysis of the indicators of the decline stage according to the relevant laws of volleyball. We should also find out the existing related problems and take corresponding measures to prevent and cure, so as to make volleyball get long-term development. The main reasons for errors in the second half of the prediction curve include the following: (1) the optimization model can better describe the linear stage of volleyball sports indicators, but there are still deficiencies in the description of the falling stage, which is also our next research direction, and (2) the optimization model has a good performance in Figure 12, while the latter half of Figure 13 has a poor performance, because the data in Figure 13 are predicted values, which have certain influence and uncertainty on the model, leading to errors.

## 5. Conclusion

1. With the increase of response, the beam change curves of different algorithms show multiple fluctuation changes in which the fluctuation range of MPDR algorithm is large. The curve obtained by MVDR algorithm is close to the fluctuation characteristics of the optimization algorithm on the whole. The beam changing pattern obtained by the optimization algorithm can better reflect the changing trend of the beam.
2. Fuzzy neural network and simulation cluster analysis method are used to analyze volleyball, and the overall analysis curve of data clustering performance under different parameters shows a gradual downward trend. The variation patterns of A2 and A3 curves are basically the same, showing a slow decline. A4 shows a step downward trend, A1's downward trend is similar to the change trend of logarithmic function, and the curve corresponding to A5 experiences a drop in a short period of time.
3. As the number of iterative steps increases, the data described by the original model can be divided into linear increase stage, slow increase stage, and fluctuation stage. The original model can only describe the first stage of volleyball. The optimization model can not only better analyze the first stage of volleyball but also better characterize its second and third stages.

## Figures and Tables

**Table 1:** Comparison of different neural networks.

Neural network	Arithmetic	Number of neurons	DA/%	*P*
NN1	OLS	77	95.13	6.84
	FRA	76	95.52	6.51

NN2	OLS	104	94.32	7.13
	FRA	102	94.76	6.78

NN3	OLS	83	96.47	7.89
	FRA	81	96.98	7.73

**Table 2:** Attribute distribution table.

Overall classification	Variable	Minimum	Maximum	Average	Median
Nuclear structure	Average degree	0.32	4.17	2.25	2.13
	Average weighting	0.14	10.23	5.18	5.13
	Average path length	1.38	3.47	2.43	2.37
	Grid density	0.36	0.74	0.55	0.52
	Mean clustering coefficient	0.00	0.42	0.21	0.19

Secondary structure	Average degree	1.43	5.12	3.28	3.25
	Average weighting	2.14	3.45	2.80	2.69
	Average path length	1.78	3.49	2.64	2.71
	Grid density	0.12	0.78	0.45	0.43
	Mean clustering coefficient	0.00	0.43	0.22	0.26

## Data Availability

The data used to support the findings of this study are available from the corresponding author upon request.
